# Macular Telangiectasia type 1 treated with integrated traditional Chinese acupuncture and Conbercept: a case report

**DOI:** 10.3389/fmed.2026.1717145

**Published:** 2026-04-30

**Authors:** Zhifei Liu, Conghui Liu, Siyue Yang, Xiaowei Tang, Jiajia Wang, Jieqiong Zhang

**Affiliations:** 1 Department of ENT, Ophthalmology and Oral Medicine, Chinese PLA 76th Army Corps Hospital, Xining, China; 2 Department of Ophthalmology, Air Force Hospital of Western Theater Command, PLA, Chengdu, China

**Keywords:** case report, Conbercept, integrated Chinese and Western medicine, Macular Telangiectasia type 1, traditional Chinese acupuncture

## Abstract

**Introduction:**

Macular Telangiectasia type 1 is a congenital vascular disorder primarily affecting males, characterized by unilateral exudative lesions on the temporal macula. Intravitreal anti-VEGF therapy is commonly used, but the response can be suboptimal in some patients. In this case, we report the management of Macular Telangiectasia type 1 using a integration of intravitreal anti-VEGF therapy and traditional Chinese acupuncture.

**Case presentation:**

A 38-year-old male presented with progressive vision loss and metamorphopsia in his right eye. Fundus examination and multimodal imaging (OCT/OCTA and fluorescein angiography) identified a perimacular hard exudate ring, cystoid macular edema, and juxtafoveal telangiectasis, consistent with idiopathic Macular Telangiectasia type 1. For the intervention, the patient received a total of three intravitreal Conbercept injections administered once monthly, integrated with peri-treatment acupuncture at predefined acupoints. Four months after the last treatment, the patient exhibited both visual and anatomical improvement.

**Conclusion:**

The integration of traditional Chinese acupuncture with Conbercept may offer therapeutic benefits for Macular Telangiectasia type 1 in this patient. Clinically meaningful improvements were observed, including improved visual acuity, reduced metamorphopsia, and decreased central retinal thickness over follow-up.

## Introduction

Macular Telangiectasia, initially described by Gass and Oyakawa in 1982, comprises a group of rare diseases characterized by unexplained juxtafoveal retinal telangiectasia and spontaneous capillary dilation in the macula (1, 2). Yannuzzi and colleagues categorized it into three types based on optical coherence tomography (OCT) findings, calling it idiopathic Macular Telangiectasia. Macular Telangiectasia type 1 (Mac Tel type 1) is a congenital or developmental vascular disorder predominantly affecting males unilaterally, characterized by exudative lesions resulting from telangiectasia on the temporal aspect of the macula. Visual impairment commonly results from cystoid macular edema (CME) and associated juxtafoveolar microvascular abnormalities, including perifoveal capillary dilation and microaneurysms (MAs). Crystalline deposits are typically observed in the macula and occasionally in the retinal periphery, suggesting that Mac Tel type 1 may be part of the broader spectrum of Coats disease (3, 4). Current concepts suggest that vascular changes may begin in the deep capillary plexus within the inner nuclear layer and later involve the superficial plexus. However, the pathogenesis and an optimal treatment strategy remain incompletely defined. Laser photocoagulation and intravitreal therapies, including anti-VEGF agents and triamcinolone, have been used with variable success in reducing CME (5, 6).

We report a patient with Mac Tel type 1 treated with an integration of intravitreal Conbercept and traditional Chinese acupuncture, which, to our knowledge, has not been previously described.

### Case description

**Figure 1:**
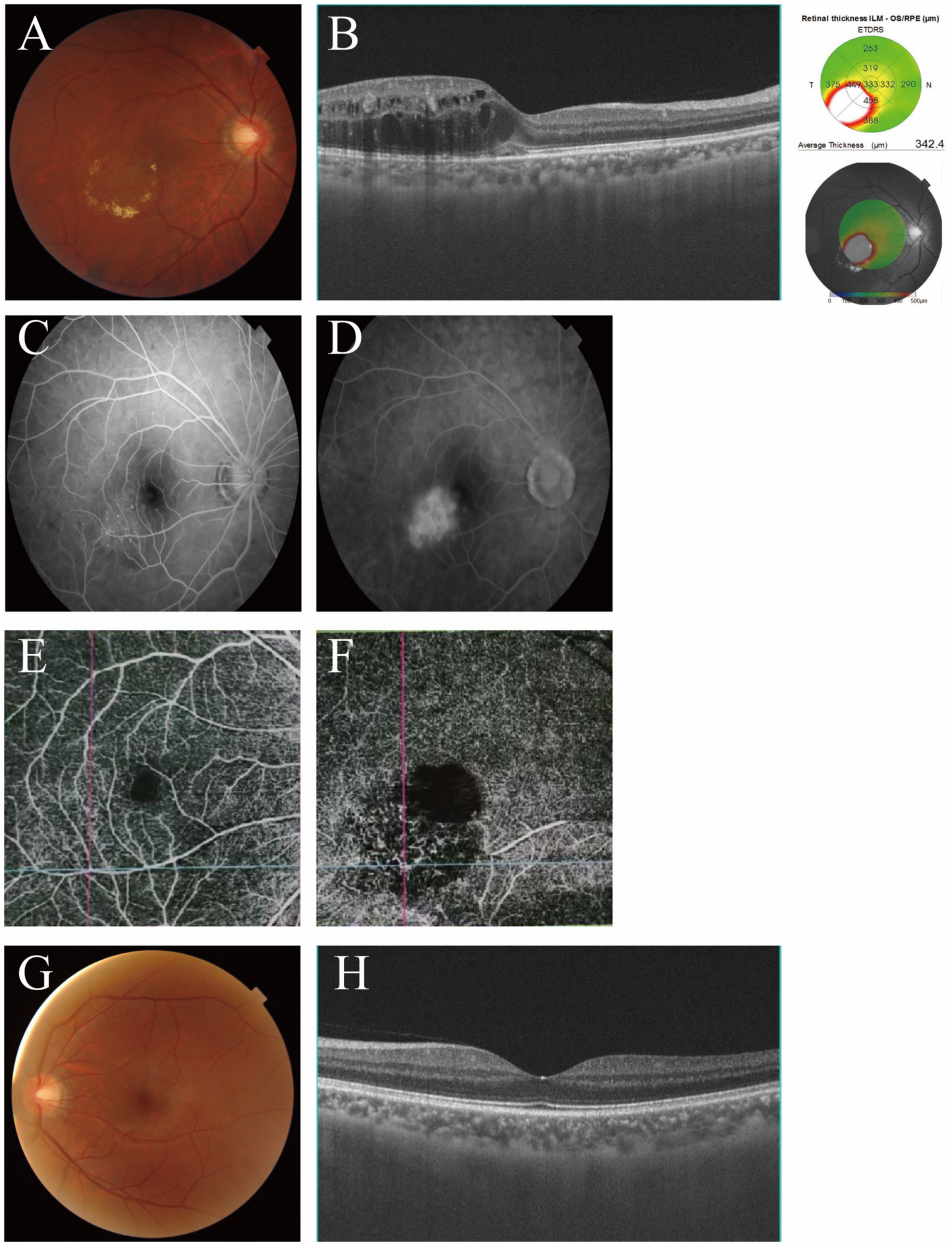
Color fundus photography of the 38-year-old patient in the right eye **(A)**, OCT **(B)**, FFA **(C,D)**, OCTA **(E,F)**; color fundus photography of the 38-year-old patient in the left eye **(G)**, OCT **(H)**.

A 38-year-old Chinese male was referred to the ophthalmology department at the Air Force Hospital of the Chinese People’s Liberation Army Western Warzone due to CME in the right eye. The patient reported experiencing progressive vision loss and metamorphopsia in the right eye for approximately eight years, with a noted deterioration four months prior to presentation. The patient had no prior history of medication for ocular discomfort before presenting to our hospital. His medical history was notable for hypertension, which was managed with a daily dose of 10 mg of amlodipine besylate. There was no history of diabetes, exposure to X-ray radiation, or retinal vein occlusion. Furthermore, his family history was unremarkable for significant ocular disorders or systemic diseases. The patient’s best-corrected visual acuity (BCVA) was 20/50 in the right eye and 20/12.5 in the left eye. Slit-lamp examination of the anterior segment in both eyes revealed no abnormalities in the conjunctiva, cornea, pupil, lens, and iris. Intraocular pressure (IOP) was 18 and 17 mmHg in the right and left eyes, respectively. Fundus examination was performed using a 90 diopter (D) Volk lens with a slit lamp after pupillary dilatation with 1% tropicamide eye drops. No vitritis was observed in either eye. The retina of both eyes was flat, with normally coursing blood vessels and a clearly demarcated, light-red optic disc. A ring of yellow exudates was visible inferior to the temporal macula in the right retina. The left retina and the remainder of the right retina showed no obvious abnormalities. Fundus color photography revealed circinate hard exudates measuring 2 disc diameters in the temporal region at the edge of the macular area, located 0.5 disc diameter from the foveola in the right eye, along with visible perimacular elevation (Figure 1A), Spectral-domain optical coherence tomography (SD-OCT) demonstrated severe cystoid macular edema (Figure 1B), with central macular thickness recorded at 342.4 μm in the right eye. In the right eye, fundus fluorescein angiography (FFA) demonstrated multiple regions of saccular hyperfluorescence located temporal to the foveal zone during the early phase, followed by leakage from these saccular structures in the late phase (Figures 1C,D). Optical coherence tomography angiography (OCTA) of the right eye revealed a slight reduction in the capillary network density of the superficial retinal layer, accompanied by significant telangiectasia of saccular capillaries with associated distortion (Figures 1E,F). In contrast, the results indicated an absence of clinically significant abnormalities in the left eye, as illustrated in Figures 1G,H. Fundus examination, along with FFA and OCTA imaging, confirmed the diagnosis of Macular Telangiectasia type 1 in the right eye.

### Therapeutic intervention

**Figure 2:**
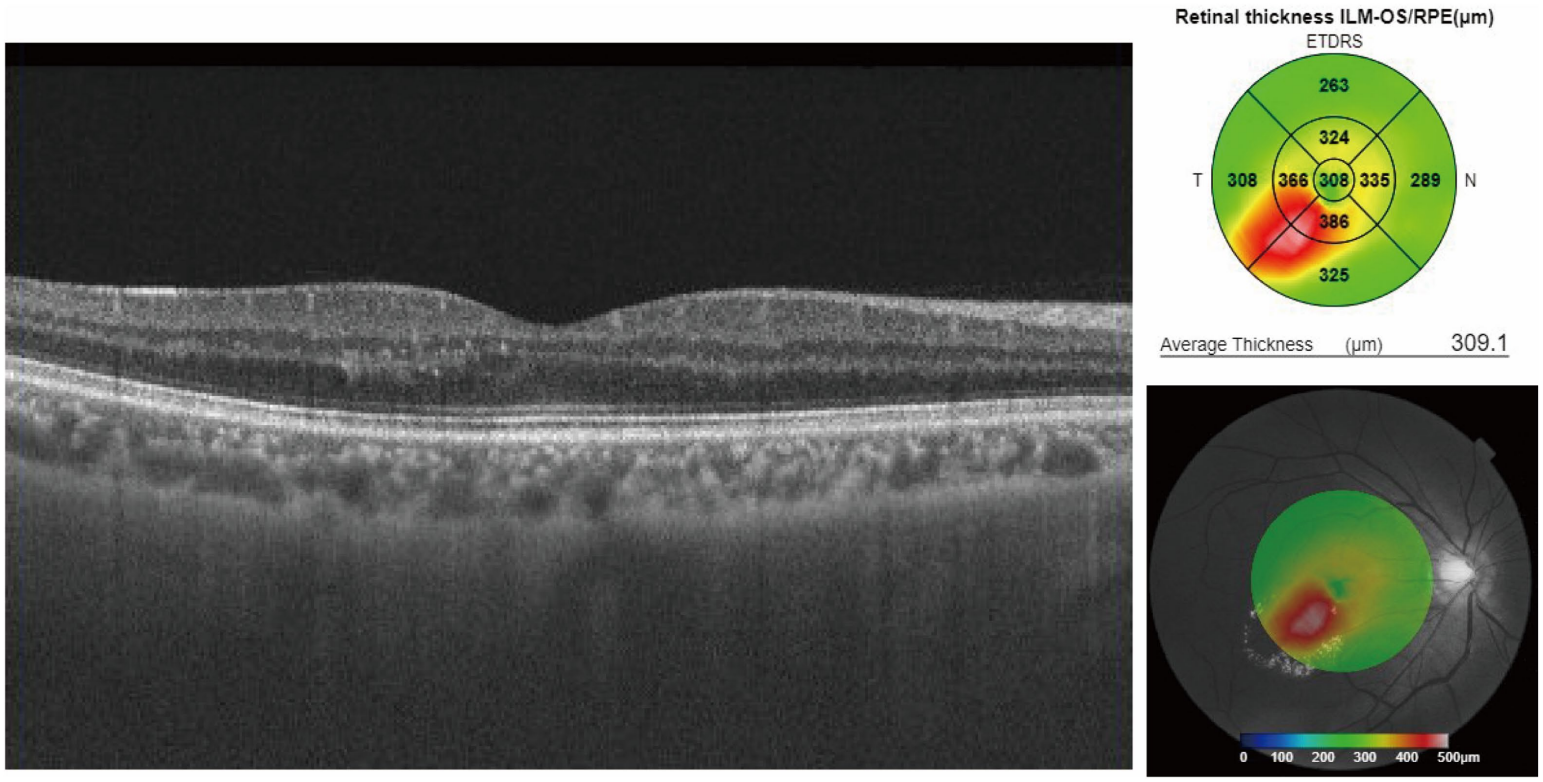
OCT image of the 38-year-old patient, taken at the one-month follow-up after the final treatment with anti-VEGF (Conbercept) and traditional Chinese acupuncture.

**Figure 3:**
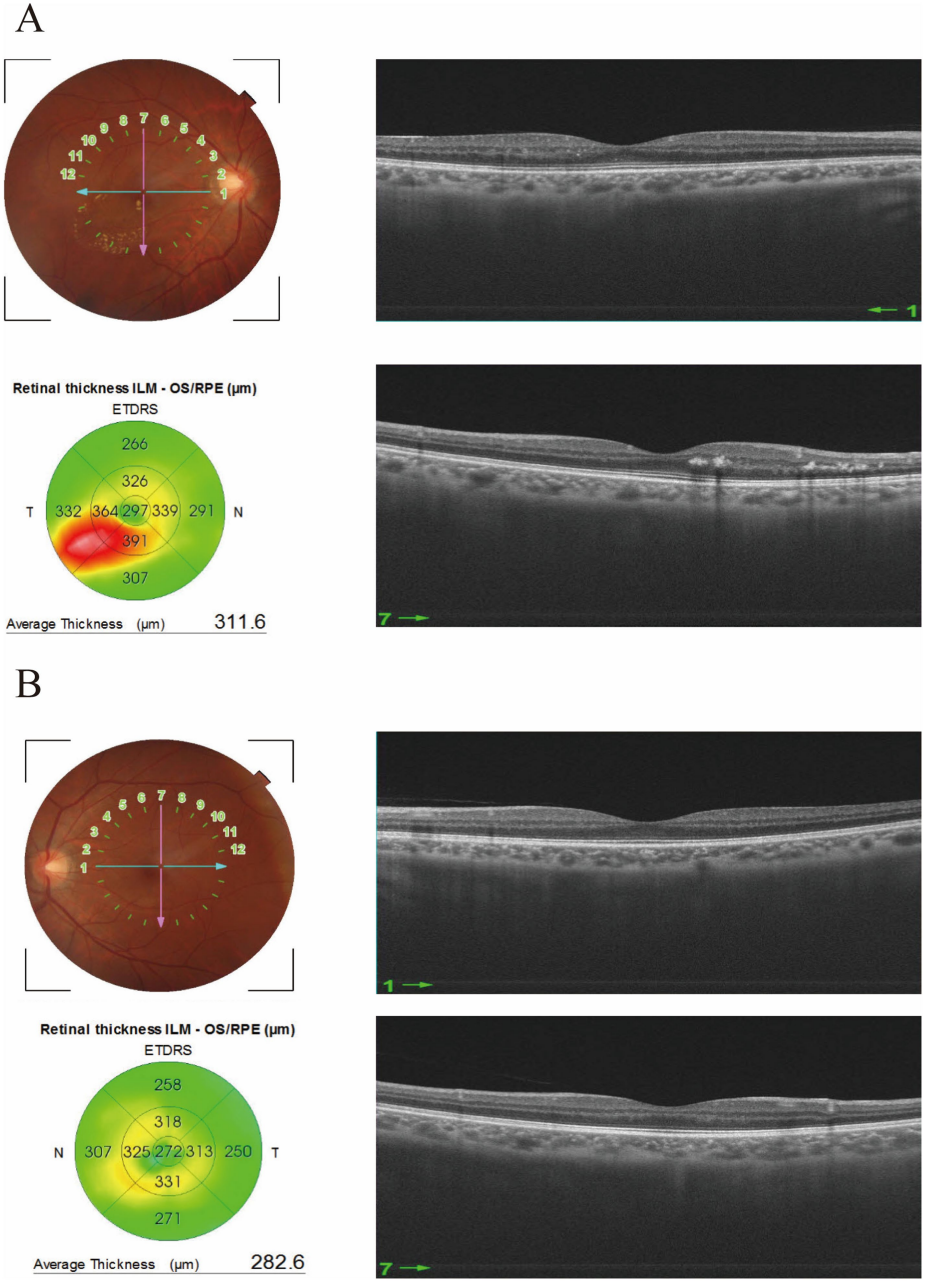
OCT images of the patient were obtained during a follow-up examination conducted four months after the final treatment. **(A)** Right eye; **(B)** left eye.

Acupuncture details were reported according to STRICTA. The patient underwent an integrated treatment of intravitreal anti-vascular endothelial growth factor (VEGF) injections and traditional Chinese acupuncture. He received three monthly intravitreal injections of 0.05 mL (0.5 mg) Conbercept in the right eye. Prior to each injection, an experienced acupuncturist administered a course of electroacupuncture, and the intravitreal injection was performed on the day following the fifth day of electroacupuncture. Electroacupuncture was administered by a licensed acupuncturist with years of clinical experience. Before each intravitreal injection, the patient received acupuncture once daily for five consecutive days, with each session lasting 30 min. Electroacupuncture (EA) was applied only on the right side at the periocular acupoints Qingming (BL1), Cuanzhu (BL2), Sibai (ST2), Yangbai (GB14), and Ashi, using a continuous wave at 1 Hz and 1.5 mA. The remaining acupoints were needled without electrical stimulation, including Guangming (GB37) on the right side and Yanglao (SI6), Yanglingquan (GB34), and Qiuxu (GB40) bilaterally. A 0.25 × 25-mm needle was inserted singly at each listed point and retained for 30 min. The electroacupuncture stimulation was delivered at 1 Hz and 1.5 mA using a continuous wave. Upon re-examination one month following the administration of three anti-VEGF injections and acupuncture therapy, the patient’s visual acuity in the right eye improved to 20/20. OCT of the right eye revealed significant resorption of macular edema, with a reduction in central macular thickness to 309.1 μm in the right eye (Figure 2). No ocular/systemic adverse events were observed. The patient also reported that the symptom of metamorphopsia significantly decreased. At the latest follow-up examination after 4 months of the combinational treatment, the patient’s visual acuity had improved to 20/12.5 in the right eye. In addition, the central macular thickness on OCT remained relatively stable in the right eye. No obvious macular abnormality was observed on OCT in the left eye (Figure 3).

## Discussion

Macular Telangiectasia type 1 (Mac Tel type 1) is a rare and potentially vision-compromising retinal vascular disorder predominantly affecting male patients. It is characterized by focal, exudative dilations of perifoveal retinal capillaries. Historically, this condition has been described by various terminologies: Leber referred to it as “miliary aneurysms,” Gass and Blodi classified it as “idiopathic juxta foveal telangiectasis” (group 1A-1B), Yannuzzi and colleagues termed it “Type 1 aneurysmal telangiectasia,” and it has more recently been designated as Mac Tel type 1 by the Mac Tel Study Group (1, 7). All classifications indicate that Macular Telangiectasia Type 1 mainly affects young males and is typically characterized by unilateral exudative telangiectasia, akin to a localized form of Coats’ disease in the macular region (3).

Currently, there is no established treatment for Macular Telangiectasia. To date, anti-VEGF agents such as bevacizumab and aflibercept have been employed to resolve cystoid macular edema (CME) in most cases. However, the emergence of drug- resistant cases necessitates the development of novel therapeutic approaches. Mitsuko Nakai and colleagues investigated the efficacy of focal photocoagulation and observed that, although microaneurysms and central retinal thickness were reduced, BCVA did not show significant improvement from baseline. This outcome may be attributed to the thermal damage inflicted on the photoreceptors of the outer retina by photocoagulation, which is particularly detrimental near the fovea. In light of this, Yong Koo Kang documented a case involving the use of a 577 nm subthreshold micropulse yellow laser (SMYL) for the treatment of macular edema associated with Macular Telangiectasia Type 1. Unfortunately, persistent CME and repeated SMYL applications may lead to atrophic changes (8, 9).

**Figure 4:**
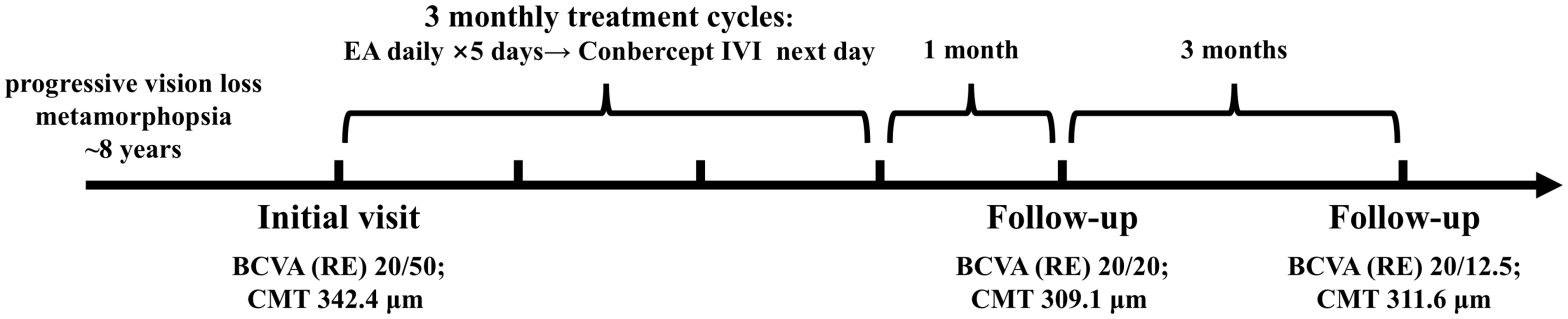
Timeline, follow-up, and clinical outcomes. BCVA, best-corrected visual acuity; CMT, central macular thickness; EA, electroacupuncture; IVI, intravitreal injection; RE, right eye.

In China, Conbercept is widely utilized as an anti-VEGF agent for neovascular age-related macular degeneration and various other retinal vascular diseases. Although Mac Tel type 1 is not an approved indication, anti-VEGF therapy is often considered for macular edema secondary to retinal vascular disorders, in line with clinical practice guidelines (10). In this case, the dose and injection interval of Conbercept were selected according to the product’s prescribing information and were consistent with the regimen evaluated in the phase 3 PHOENIX trial (11). This approach was intended to ensure that the treatment regimen was clinically appropriate and aligned with existing safety data (Figure 4).

Acupuncture, a traditional Chinese medical practice with a long history, has been extensively employed in the treatment of various diseases (12). In this case, the acupuncture protocol was integrated with anti-VEGF treatment, and acupoint selection followed a “local–distal” strategy (13). Periocular acupoints, namely Qingming (BL1), Cuanzhu (BL2), Sibai (ST2), Yangbai (GB14) and Ashi points, were selected on the affected side for local treatment, with the aim of improving periocular circulation (14, 15). Distal points, including Guangming (GB37), Yanglao (SI6), Yanglingquan (GB34), and Qiuxu (GB40), were added as supportive points according to the “local–distal” strategy. Given that the disease was unilateral, periocular stimulation was limited to the right side, whereas some distal points were used bilaterally.

From a modern medical perspective, the periocular acupoints used in this case are located close to branches of the trigeminal nerve, such as the supraorbital and infraorbital nerves. A prior study reported short-term changes in retrobulbar circulation after electroacupuncture (EA), including increased blood-flow velocity in the central retinal artery and short posterior ciliary arteries (16). Beyond hemodynamic effects, experimental studies have reported that EA can downregulate proinflammatory cytokines such as TNF-α and IL-1β and modulate angiogenesis-related signaling, including VEGF-related pathways (17, 18). This is relevant because responses to corticosteroids in some reports suggest that inflammation may contribute to exudation and CME in Mac Tel type 1 (6). These findings provide a tentative biological rationale for integrating acupuncture with anti-VEGF treatment.

This case suggests that integrating Conbercept with acupuncture may be beneficial for CME associated with Mac Tel type 1. The patient’s BCVA improved from 20/50 to 20/12.5, along with relief of metamorphopsia and anatomical improvement on OCT. The primary contribution of this report lies in its detailed description of an innovative integrative therapy combining Chinese and Western medical practices. This integrative approach may broaden the therapeutic options for CME associated with Mac Tel type 1. Several limitations should be acknowledged. As a single uncontrolled case, the observed improvement cannot be clearly distinguished from the spontaneous variation of the disease. In addition, the additional contribution of acupuncture beyond anti-VEGF therapy alone cannot be determined. Follow-up was limited to 4 months after the final treatment (7 months from the initial visit), which is insufficient to assess recurrence and long-term safety. Further studies with larger cohorts and longer follow-up are needed to confirm efficacy and establish a reproducible treatment protocol.

## Patient perspective

The patient indicated a perceived enhancement in visual acuity and a reduction in metamorphopsia, expressing satisfaction with the integrated treatment. He expressed willingness to continue follow-up.

## Data Availability

The original contributions presented in the study are included in the article/supplementary material, further inquiries can be directed to the corresponding author/s.
